# rs5888 Variant of *SCARB1* Gene Is a Possible Susceptibility Factor for Age-Related Macular Degeneration

**DOI:** 10.1371/journal.pone.0007341

**Published:** 2009-10-05

**Authors:** Jennyfer Zerbib, Johanna M. Seddon, Florence Richard, Robyn Reynolds, Nicolas Leveziel, Pascale Benlian, Patrick Borel, Josué Feingold, Arnold Munnich, Gisèle Soubrane, Josseline Kaplan, Jean-Michel Rozet, Eric H. Souied

**Affiliations:** 1 Creteil University Eye Clinic, Faculte de Medecine Henri Mondor, Creteil, France; 2 Genetics Service, INSERM U781, Hopital Necker Enfants Malades, Paris, France; 3 Tufts University school of Medicine and Ophthalmic of Epidemiology and Genetics Service, Tufts Medical Center, Boston, Massachusetts, United States of America; 4 Université Lille Nord de France, INSERM, UMR744, Institut Pasteur de Lille, Lille, France; 5 Universite Pierre et Marie Curie, Paris 6, Department of Molecular Biology and Biochemistry, Hopital Saint-Antoine, Paris, France; 6 INRA, UMR1260 « Nutriments Lipidiques et Prévention des Maladies Métaboliques », Marseille, France; 7 Unite Fonctionnelle de Recherche Clinique, Creteil, France; University of Illinois at Champaign-Urbana, United States of America

## Abstract

Major genetic factors for age-related macular degeneration (AMD) have recently been identified as susceptibility risk factors, including variants in the *CFH* gene and the *ARMS2 LOC387715/HTRA1locus*. Our purpose was to perform a case-control study in two populations among individuals who did not carry risk variants for *CFHY402H* and *LOC387715 A69S (ARMS2)*, called “study” individuals, in order to identify new genetic risk factors. Based on a candidate gene approach, we analyzed SNP rs5888 of the *SCARB1* gene, coding for SRBI, which is involved in the lipid and lutein pathways. This study was conducted in a French series of 1241 AMD patients and 297 controls, and in a North American series of 1257 patients with advanced AMD and 1732 controls. Among these individuals, we identified 61 French patients, 77 French controls, 85 North American patients and 338 North American controls who did not carry the *CFH* nor *ARMS2* polymorphisms. An association between AMD and the *SCARB1* gene was seen among the study subjects. The genotypic distribution of the rs5888 polymorphism was significantly different between cases and controls in the French population (p<0.006). Heterozygosity at the rs5888 SNP increased risk of AMD compared to the CC genotypes in the French study population (odds ratio (OR) = 3.5, CI95%: 1.4–8.9, p<0.01) and after pooling the 2 populations (OR = 2.9, 95% CI: 1.6–5.3, p<0.002). Subgroup analysis in exudative forms of AMD revealed a pooled OR of 3.6 for individuals heterozygous for rs5888 (95% CI: 1.7–7.6, p<0.0015). These results suggest the possible contribution of *SCARB1*, *a* new genetic factor in AMD, and implicate a role for cholesterol and antioxidant micronutrient (lutein and vitamin E) metabolism in AMD.

## Introduction

Age-related macular degeneration (AMD) is the most common cause of irreversible vision loss in the elderly population in Europe and United States [1], [2]. Identification of risk factors is of major importance for the understanding of the origins of the disorder and for establishing strategies to prevent AMD. Risk factors for AMD are both environmental [3]–[9] and genetic [10]–[14]. Over the past few years, several single nucleotide polymorphisms (SNPs) have been associated with AMD, including variants in the *CFH* and *ARMS2* genes [15]–[26], [64]. The association between these polymorphisms and AMD risk suggests a pathway of inflammation and oxidation in AMD. Besides these pathways, several lines of evidence suggest a strong role of antioxidant micronutrients (xanthophylls, vitamin E and vitamin C) and lipids in AMD [8], [27]–[33].

Our purpose was to assess candidate genes and polymorphisms involved in the lipid pathway among individuals harboring non-risk alleles for *CFH* and ARMS2. The *SCARB1* gene which encodes SRB1, a multiligand cell surface receptor, is known to mediate selective cholesterol uptake, and cholesterol efflux [34]–[37], and was regarded as an attractive candidate gene. Furthermore, recent studies have shown that *SCARB1* is also involved in uptake of vitamin E and lutein giving further support to a possible role of *SCARB1* in AMD [38], [39]. In fact, the xanthophyll, lutein, is recovered at high concentration in the human macula lutea and has been associated with the risk of AMD, and vitamin E, the main lipophilic antioxidant, is suspected to prevent oxidation of polyunsaturated fatty acids recovered at high concentrations in the human retina [40]–[42]. Several studies have suggested that *SCARB1* genotypes for the rs5888 synonymous SNP may play a role in cholesterol homeostasis and is associated with cardiovascular diseases [43]–[48]. Herein, we report a possible association between the *SCARB1* rs5888 SNP and AMD in a subgroup of French and North American AMD patients in a case-control study.

## Results

The French cohort consisted of 1241 cases (92% exudative AMD, 5.2% geographic atrophy, 2.8% early or intermediate AMD) and 297 controls. The mean±SD age at AMD diagnosis was 78.8±7.5 years. The North American cohort consisted of 1732 advanced AMD cases (72.4% exudative AMD, 27.6% geographic atrophy) and 1257 controls. All subjects were Caucasian. The mean±SD age at AMD diagnosis was 80.3±6.5 years (Table 1).

**Table 1:** Non-genetic characteristics of the entire French and North American populations.

	French population			USA population		
	Controls	Cases		Controls	Cases	p
**N**	**297**	**1241**		**1257**	**1732**	
**Sex, men, n(%)**	110 (39.4%)	415 (33.4%)	≤0.06	543 (43.2%)	750 (43.3%)	<0.96
Age, m (sd)*	69.2 (7.4)	78.8 (7.5)	<0.0001	75.0 (5.5)	80.3 (6.5)	<0.0001
**Smoking**						
** Current, n(%)**	89 (30.1%)	106 (8.6%)	<0.0001	27 (4.6%)	98 (7.9%)	<0.005
** Never, n(%)**	163 (55.1%)	770 (62.1%)		251 (42.9%)	455 (36.7%)	
** Past, n(%)**	44 (14.8%)	364 (29.3%)		307 (52.5%)	686 (55.4%)	

The genotype distributions of the rs1061170, rs10490924 and rs5888 SNPs within the *CFH*, *ARMS2* and *SCARB1* genes, respectively, are shown in Table 2. The genotypic distributions of the *CFH Y402H* and *ARMS2* SNPs were significantly different between cases and controls in both the French and North American series (p<0.0001 for both groups). For rs5888 genotypes, regardless of *CFH* or *ARMS2* genotypes, no significant association was found in the entire French population of AMD patients (Table 2). However, the distribution of the *SCARB1* rs5888 genotype was significantly different in the North American AMD population compared to controls (p<0.004): CT heterozygotes were at increased risk of AMD compared to CC subjects (adjusted OR_CT vs CC_ = 1.4, 95%CI 1.0–1.8), TT did not significantly differ from CC (adjusted OR = 1.2 CI95% 0.9–1.7).Similar results were obtained after pooling the French and the North American population: adjusted OR_CT vs CC_ = 1.3, 95%CI: 1.0–1.7) and adjusted OR_TT vs CC_ = 1.2, 95%CI 0.9–1.7. Odds Ratio were adjusted for non genetic confounders: age, gender and smoking status.

**Table 2:** Genotype distributions among the entire French and North American populations.

	French population			USA population		
	Controls	Cases		Controls	Cases	p
**N**	**297**	**1241**		**1257**	**1732**	
***CFHY402H (rs1061170)***						
**CC**	35 (11.8%)	356 (28.7%)	**<0.0001**	154 (12.3%)	654 (37.8%)	**<0.0001**
**CT**	146 (49.2%)	628 (50.6%)		562 (44.7%)	815 (47.1%)	
**TT**	116 (39.0%)	257 (20.7%)		541 (43.0%)	263 (15.2%)	
**ARMS2 (rs10490924)**						
**GG**	195 (65.7%)	397 (32.0%)	**<0.0001**	799 (63.6%)	542 (31.3%)	**<0.0001**
**GT**	92 (31.0%)	577 (46.5%)		416 (33.1%)	814 (47.0%)	
**TT**	10 (3.4%)	267 (21.5%)		42 (3.3%)	376 (21.7%)	
**SRB1 (rs5888)**						
**CC**	79 (26.6%)	317 (25.5%)	**<0.89**	376 (29.9%)	433 (25.0%)	**<0.004**
**CT**	151 (50.8%)	629 (50.7%)		585 (46.5%)	903 (52.1%)	
**TT**	67 (22.6%)	295 (24.8%)		296 (23.6%)	396 (22.9%)	

P values: global chi^2^ test with 2 degrees of freedom for comparison of genotype distribution between cases and controls.

Characteristics of subjects carrying no risk alleles at the *CFH* and ARMS2 loci are shown in Table 3 (and Table S1). In the French population, genotypic distribution of the *SCARB1* polymorphism was significantly (p<0.01) different between cases and controls: CT heterozygotes compared to CC subjects were at increased risk of AMD. (adjusted OR 3.5, 95%CI1.4–8.9). In the North American population, we observed a suggested increased risk of AMD associated with heterozygocity at the rs5888 (global test p<0.09): OR_CT vs CC_ = 2.5; 95%CI 1.1–5.7. Similar results were obtained when pooling French and North American populations (global test, p<0.002), in that CT individuals were at increased risk of AMD compared to CC genotypes: adjusted OR = 2.9; 95%CI1.6–5.3 while TT subjects did not significantly differ from CC subjects (Table 4).

**Table 3:** Non-genetic characteristics of the French and North American populations with no risk alleles for *CFH* and *ARMS2*.

	French population			USA population		
	Controls	Cases		Controls	Cases	p
**N**	**77**	**61**		**338**	**85**	
**Sex, men, n(%)**	23 (31.9%)	16 (26.2%)	**<0.48**	144 (42.6%)	39 (45.9%)	**<0.59**
Age, m (sd)*	70.3 (7.2)	77.9 (9.8)	**<0.0001**	74.8 (5.6)	79.0 (8.0)	**<0.0001**
**Smoking**						
** Current, n(%)**	19 (24.7%)	10 (16.4%)	**<0.50**	8 (5.0%)	8(14.3%)	<0.062*
** Never, n(%)**	46 (59.7%)	41 (67.2%)		72 (45.0%)	20 (35.7%)	
** Past, n(%)**	12 (15.6%)	10 (16.4%)		80 (50.0%)	28 (50.0%)	

**Table 4:** Adjusted Odds ratio *for rs5888 of SCARB1 gene* in patients with no risk alleles for *CFH* and *ARMS2*.

	CC	CT	TT	
		OR [CI95%]	OR [CI95%]	P*
**France**	1 (ref)	3.5 [1.4–8.9]	1.0 [0.3–3.2]	**<0.01**
**USA**	1 (ref)	2.5 [1.1–5.7]	2.0 [0.8–5.2)	**<0.09**
**Pooled**	1 (ref)	2.9 [1.6–5.3]	1.6 [0.8–3.3]	**<0.002**
**Gender-Pooled**				
**Men**	1 (ref)	4.3 [1.5–11.9]	1.8 [0.5–6.3]	**<0.02**
**Women**	1 (ref)	2.5 [1.2–5.5]	1.7 [0.7–4.3]	**<0.07**
**Exudative forms**				
**France**	1 (ref)	3.6 [1.3–10.0]	1.1 [0.3–4.0]	**<0.02**
**USA**	1 (ref)	3.5 [1.1–10.5]	2.2 [0.6–8.0]	**<0.09**
**Pooled**	1 (ref)	3.6 [1.7–7.6]	1.6 [0.7–4.0]	**<0.0015**

Adjustment for non genetic confounders: age, sex, and cigarette smoking.

For table 4, OR are estimated by genotype (CT vs CC and TT vs CT) but the p values are global p values (with 2 degrees of freedom) for estimates a global effect of genotype (is at least one of the genotypes (CT or TT) significantly associated with increased rik of AMD).

*Interaction with center; p<0.29, interaction with sex (in pooled population), p<0.48.

Sample size: France: 61 cases and 72 controls – USA: 56 cases and 160 controls.

In exudative AMD subgroups (Cases: n = 105, 55 French and 50 American; controls: n = 415, 77 French and 338 American), adjusted OR after pooling both populations for CT heterozygous individuals was: OR = 3.6, 95%CI: 1.7–7.6, p<0.0015.

## Discussion

Here we report for the first time a possible association between a polymorphism in the *SCARB1* gene and AMD, in two distinctly different Caucasian populations.

AMD is a multifactorial disorder including both environmental and genetic factors. Among the long list of genes potentially involved in AMD [49], two major genes have been recently associated to AMD: *CFH* and *ARMS2* [15]–[26], [64]. Because, double homozygosity for the *CFH* and *ARMS2* risk alleles account for more than 50% in the pathology of AMD [50], [51], we hypothesized that candidate gene screening in a subgroup of AMD patients and controls homozygous for *CFH* and *ARMS2* wild-type alleles may help in identifying novel and independent genetic risk factors. One limit of our study is the sample size of our populations without the CFH and/or the ARMS2 at-risk alleles. Only 61/1241 (4.9%) of AMD French patients and 85/1732 (4.9%) of American AMD did not carry one of these at-risk alleles. Thus, it requires large series of patients in order to assure that some patients without the CFH and/or the ARMS2 at-risk alleles are present in the final sample.

Heterozygosity for the rs5888 SNP of the *SCARB1* gene (CT) may be associated with an increased risk of AMD in the French and North American populations, respectively. Nevertheless, our findings have to be interpreted very cautiously. Heterozygosity was found significantly associated to AMD in wild-type individuals for *CFH* and ARMS2 in the French group (p<0.01), and in the same direction but not significant in the North American population (p<0.09). Heterozygosity at the rs5888 was also found to be significantly associated with AMD when all North American individuals, regardless of their genotypes at the *CFH* and *ARMS2* loci, were considered (p<0.004), but not in the French population. This discrepancy might be explained by the low number of French controls, compared to the North-American sample. However the number of French AMD patients and controls (respectively 1241 and 297) was evaluated in order to obtain similar number of AMD patients and controls in wild-types groups with no risk alleles for CFH and *ARMS2* (respectively 61 and 77). An association between rs5888 of *SCARB1* and the exudative type of AMD was observed (OR: 3.6, 95%CI: 1.7–7.6; p<0015). We enrolled a large number of exudative forms of AMD because patients with neovascular AMD are most often referred to our specialized retina departments than atrophic forms of AMD.

*SCARB1* gene is located in 12q24.31, in a region of interest pointed by linkage analysis [52], [53]. *SCARB1* gene encodes a multiligand cell surface receptor that mediates selective cholesterol uptake, and cholesterol efflux [34]–[37]. Reverse cholesterol transport, is a major pathway for the clearance of excess cholesterol from the body. Several studies have reported that the *SCARB1* rs5888 SNP is associated with the development of coronary heart disease [44], and lipid profile [45]–[48]. Indeed, epidemiological studies in Caucasian populations have shown that the rs5888 is associated with increased HDL cholesterol and lower LDL cholesterol, and rs5888 has been reported to be associated with a greater risk of developing coronary heart disease in males [44]. Because AMD and cardiovascular diseases share common pathways [54]–[56], we decided to analyze genes involved in lipid homeostasis. Furthermore, it is known that *SCARB1* is also expressed in the retinal pigment epithelium [57], and could interact with APOE, another gene which some groups but not all have reported to be involved in AMD [58], [59]. Besides the pathway of lipids, *SCARB1* is also involved in the metabolism of lutein and vitamin E [60]. Lutein, obtained from foods, is a member of the carotenoid family, more specifically the xanthophyll family. Lutein protects the photoreceptors against light-initiated oxidative damage. Furthermore, epidemiologic studies based on diet questionnaires or serum levels of lutein revealed that high levels of lutein are associated with a decreased risk of AMD [8], [61]. Vitamin E acts as an antioxidant, protecting the retina against oxidative stress, with possible preventive and therapeutic effects [33], [40], [41]. *SCARB1* is involved in the metabolism of three key molecules involved in the etiology of AMD: cholesterol, lutein and vitamin E, all supported by fundamental and epidemiological studies. For these reasons based on gene function and gene location, we considered *SCARB1* as a good candidate gene for AMD. The greater risk of AMD found in CT individuals heterozygous at rs5888 has already been reported in peripheral arterial disease [43]. The rs5888 SNP is a coding-synonymous polymorphism (A350A). This polymorphism may nevertheless be in linkage disequilibrium with a functional sequence change as recently demonstrated with the identification of the rs10490924 polymorphism in the *ARMS2* gene which results in instability of the transcript [62]. To evaluate this hypothesis, we sequenced the 13 *SCARB1* exons (in 8 patients: 3 patients TT, 2 patients CC and 3 patients CT) and 200 kb of the 5′UTR in 92 wild-type individuals patients and controls, but we did not found any anomalies in the gene sequence, neither insertions or deletions such as the one described in the *SCARB1* promotor [63]. The relatively small sample size in some of the subgroup analyses could also explain some of the findings. On the other hand, it is also possible that the rs5888 polymorphism has a functional effect through a mechanism involving splicing regulatory system. From this point of view it is worth noting that it has been shown that *SCARB1* mRNA expression is significantly decreased in heterozygous individuals compared to homozygous CC or TT [43]. “A dominant-negative effect can be suggested.” Further studies will hopefully bring insights into this intriguing question.

In conclusion, our results suggest that the SCARB1 polymorphism is associated with AMD. This genetic finding is consistent with basic and epidemiological studies underlying the role of cholesterol, lutein and vitamin E in AMD. Additional studies including correlations with serum analysis and larger samples sizes are needed to confirm this finding.

## Methods

### French Populations

#### Patients

Written informed consent was obtained, as required by the French bioethical legislation and local ethic committee (CCPPRB Henri Mondor), in agreement with the Declaration of Helsinki for research involving human subjects.

A total of 1241 French AMD patients were recruited in 4 French Ophthalmologic Centres, at the Ophthalmology Eye Clinic of Créteil in collaboration with the Pellegrin Hospital, the Quinze-Vingts Hospital and the Centre of Imaging and Laser of Paris, between November 2005 and July 2007. Inclusion criteria of the AMD patients were (1) women or men aged 55 or older, and (2) with exudative AMD, atrophic AMD or with early or intermediate AMD (also called Aged-Related Maculopathy) in at least one eye. Exclusion criteria were presence of other retinal disease (*e.g.* diabetic retinopathy, high myopia, or macular dystrophies). Patients underwent a complete ophthalmologic examination including best corrected visual acuity measurement, fundus examination, and retinal photographs. Fluorescein angiography (Topcon 50IA camera, Tokyo, Japan)- and if needed indocyanine green angiography (HRA, Heidelberg, Germany)- and Optical Coherence Tomography (Carl Zeiss Meditec, Inc) were performed. A questionnaire about medical history and smoking was completed.

#### Controls

A total of 297 French women or men over 55 years with a normal fundus examination and a normal aspect of fundus photography were also recruited at the Ophtalmology Eye Clinic of Créteil between 2002 and 2008. Information about their medical history including smoking, was obtained.

#### Genotyping Methods

Genomic DNA was extracted from 10 mL blood leukocytes using the Illustra® kit according to the manufacturer protocol (GE Healthcare). The *SCARB1* rs5888 *CFH* Y402H and *ARMS2* rs10490924 SNPs were genoyped by quantitative PCR allelic discrimination using reagents and conditions from Custom Taqman SNP Genotyping Assays (Applera Corp., France), using ABI 7900HT (Applied Biosystems) [24].

### North American Populations

#### Patients

Subjects and methods of diagnosis and enrollment have been previously described [64]. All patients had advanced age-related macular degeneration, either exudative or geographic atrophy, and diagnosis was based on ocular examination and fundus photography. They were all Caucasian and unrelated (Table 1).

#### Controls

Caucasian individuals without AMD who were unrelated to the cases and to other controls were enrolled. Absence of AMD was based on ocular examination and grading of fundus photographs [64] (Table 1). All cases and controls signed a written informed consent form.

#### Genotyping Methods

DNA samples were evaluated for the rs5888 SNP using either the Affymetrix 6.0 platform as part of our genome-wide association study (under review) or the Sequenom platform. Genotyping was performed at the Broad Institute in Cambridge, MA, USA.

#### Statistical Analysis

Hardy-Weinberg assumption was assessed by the standard method comparing the observed numbers of subjects in different genotype categories with the expected number under Hardy-Weinberg equilibrium for the estimated allele frequency, and testing with a Pearson goodness-of-fit statistic with the χ^2^ with 1 degree of freedom.

χ2 test was used to compare categorical allelic and genotype distributions between cases and controls (table 1). General linear models were used to compare means between cases and controls. Logistic regression models were used to estimated odds ratio (OR) with 95% confidence interval (95%CI) for AMD risk. OR's were adjusted for age, gender and smoking status. Significance levels were set at p<0.05. Analyses were performed with the SAS software release 9.01 (SAS Institute INC, Cary, NC).

Homogeneity between the 2 populations was tested by introduction of interaction terms with study center in the models (1rst test), by Breslow-day Test for homogeneity of the odds ratio (2nd test) and by I^2^ ( =  % of heterogeneity). I^2^ has been estimated by the software Review Manager 5. I^2^ and Breslow-Day test have been made without adjustment.

## Supporting Information

Table S1 Genotype distribution for SCARB1 (rs5888) in the French and North American individuals with no risk alleles for CFH (rs1061170) and ARMS2 (rs10490924). P values: global chi^2^ test with 2 degrees of freedom for comparison of genotype distribution between cases and controls.
(0.03 MB DOC)
