# Supplementary material for: rs5888 Variant of SCARB1 Gene Is a Possible Susceptibility Factor for Age-Related Macular Degeneration
Source: PLoS One. 2009 Oct 5;4(10):e7341. doi: 10.1371/journal.pone.0007341 (PMC2752725; doi:10.1371/journal.pone.0007341)
Supplement: Table S1 — Genotype distribution for SCARB1 (rs5888) in the French and North American individuals with no risk alleles for CFH (rs1061170) and ARMS2 (rs10490924). P values: global chi2 test with 2 degrees of freedom for comparison of genotype distribution between cases and controls. (0.03 MB DOC) [file pone.0007341.s001.doc]

|  | French population |  |  | USA population |  |  |
| --- | --- | --- | --- | --- | --- | --- |
|  | Controls | **Cases** |  | Controls | **Cases** | **p** |
|  |  |  |  |  |  |  |
| **N** | **77** | **61** |  | **338** | **85** |  |
| **rs5888** |  |  |  |  |  |  |
| **Genotype frequencies** |  |  |  |  |  |  |
|  |  |  |  |  |  |  |
| **CC, n(%)** | 29 (37.7%) | 11 (18.0%) | **< 0.006** | 110 (35.5%) | 18 (21.2%) | **< 0.12** |
| **CT, n(%)** | 31 (40.3%) | 41 (67.2%) |  | 146 (43.2%) | 45 (52.9%) |  |
| **TT, n(%)** | 17 (22.1%) | 9 (14.8%) |  | 82 (24.3%) | 22 (25.9%) |  |
